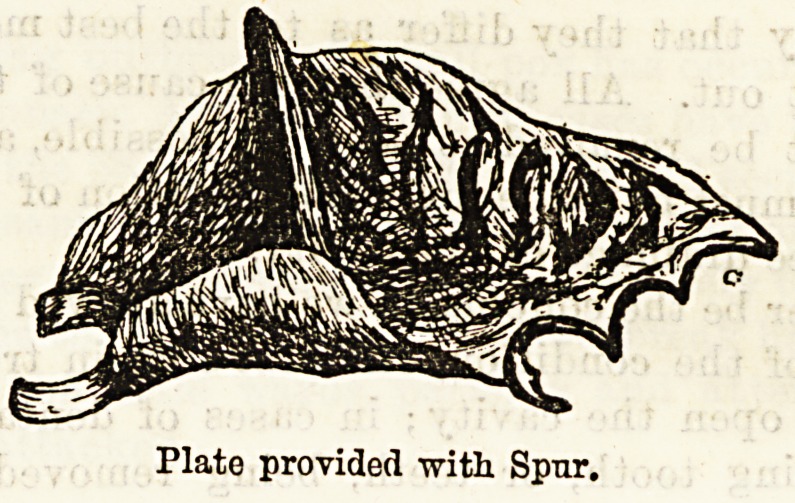# Empyema of the Antrum

**Published:** 1894-03-17

**Authors:** 


### Medical Progress and Hospital Clinics.

[*The Editor will be glad to receive offers of co-operation and contributions from members of the profession. All letters should be addressed to* The Editor, The Lodge, Porchester Square, London, W.]

EMPYEMA OF THE ANTRUM (*continued*).

It has been stated in this article that among writers on antral disease great differences of opinion exist on the subject of treatment. It would be more strictly true to say that they differ as to the best method of carrying it out. All agree that the cause of the mischief must be removed as soon as possible, and that provision must be made for the evacuation of the pus, and for free drainage of the cavity.

Whatever be the conclusion we have arrived at as to the cause of the condition, our first step in treatment will be to open the cavity; in cases of dental origin the offending tooth, or teeth, being removed at the same time.

In opening and draining the antrum we have the choice of three methods. (1) To drain through the natural orifice. (2) To perforate the inner wall of the antrum from the inferior meatus, and drain through the nose. (3) To open the antrum from the mouth, either through the alveolus, from the canine fossa, or by an opening near the first molar.

We may practically disregard the first method; it holds out but little hope of success. Apart from the difficulty of reaching the orifice, and the impossibility of the patient carrying on the treatment himself, the situation of the ostium maxillare is such that free drainage cannot be obtained.

Great difference of opinion exists as to which of the two remaining methods should be adopted. It is claimed for the operation from the inferior meatus (1) that the danger of micro-organisms and particles of food entering the antrum and keeping up irritation is obviated. It is a question whether germs are not as likely to enter from the nose. The objection as to the entrance of food would have greater weight were it not a very simple matter to exclude it. There are some very important objections to Mikulicz's operation. The cavity is much less accessible for purposes of treatment from the nose, and patients can hardly carry on the treatment themselves, a matter of considerable importance in certain circumstances. Moreover, two great sources of discomfort to the patient are not removed by this method, viz., the smell, and the discharge from the nose; pus is also more likely to be swallowed during sleep than when the antrum communicates with the mouth.

In operating from the mouth opinions differ as to the most suitable position for the opening.

Some prefer to perforate from the canine fossa, or, as advised by Mr. Heath, above the alveolus close to the first molar. Others maintain that the most suitable place is through the socket of a tooth, preferably of the first molar. The first situation offers certain advantages. When the teeth are free from disease, the sacrifice of a healthy tooth is avoided. Packing the antrum is certainly easier if the opening is made from the canine fossa. The cheek also will act as a valve closing the opening and preventing, though not entirely, the entrance of food.

When teeth must be removed, and they must be in nearly every case, the alveolar method offers the distinct advantage, that the operation may be completed at once by perforating through the socket, especially as in some cases the mere extraction of a tooth suffices to open the cavity. Drainage, too, is rather more perfect as the cavity is opened in its most dependent part. It is true that food enters more easily when the opening is situated in the alveolus, but the fact is not of great importance, as by the employment of a plate food may be perfectly excluded, the plate being of still further advantage by controlling the escape of pus, so that the patient is spared the discomfort and danger to which he is exposed when the discharge is free to flow constantly into the mouth. It is, however, impossible to curette the cavity through an opening in the alveolus.

In operating on a case of antral disease, it may be laid down as a safe rule to follow, that all roots and

Diagram showing the position of the opening from the canine fossa.

dead teeth should be removed, whether they show signs of inflammation or not. The reason for this is clear, when we consider that any tooth in the upper jaw may come into relation, directly or indirectly, with the antrum.

In perforating from the alveolus, the opening is most easily made through the socket of the anterior buccal root of the first molar. The opening may be made either by a trocar or by a large spear-headed drill used in the dental engine. The instrument must be guarded either by a metal rim or by being thrust through a good firm cork. Without this precaution it is apt to slip, and may wound the floor of the orbit. The opening so made must be enlarged with a large rose-headed drill. After puncturing through the root socket, it is a good plan to remove the septum between the three roots by twisting it away with forceps, so that an opening is made equal in size to the tooth. It is of the first importance that the opening should be a free one. Perforation from the canine fossæ may be performed with a guarded trocar or large spear-headed drill, the instrument being directed somewhat upwards to avoid wounding the floor of the antrum.

The operation can be very easily accomplished by the use of a couple of small trephines worked by the dental engine, one of them having a knife edge for division of the soft parts.

A sufficient opening having been made, the antrum must be very thoroughly washed out with warm water, to remove blood clots and masses of inspissated pus, directing the stream against the inner wall, so as to free the natural opening.

The ordinary glass syringe is useless for this purpose, though a makeshift arrangement may be made by slipping a piece of small rubber drainage tube on the nozzle.

The vulcanite syringes generally sold for the purpose, though convenient from the ease with which the shape of the nose can be altered, by oiling and warming over a spirit lamp, are too small to give a sufficient stream to thoroughly cleanse the cavity. A serviceable syringe should hold about two ounces, and have a long flexible nozzle of gum elastic.

Syringing should be carried out two or three times a day, using first some mild antiseptic lotion; a saturated solution of boracic acid or boro-glyceride in water, being the most suitable. After a time something more stimulating and astringent will be necessary. Weak solutions of nitrate of silver, or sulphate of zinc, two or three grains to the ounce, or a weak solution of tincture of iodine in water may be used. Listerine, one in twenty, makes a very agreeable injection. Chloride of zinc, three, five, and in very chronic cases, ten grains to the ounce of water, is an excellent lotion, and removes the fætor sooner than any other application.

There are two important points to remember in the use of injections: (1) They must be frequently changed, as the mucous membrane becomes rapidly accustomed to any particular drug. (2) They must always be used warm; the injection of cold fluid will almost certainly keep up irritation and retard healing. Lightly packing the antrum with a narrow strip of gauze soaked in one of the above lotions, is very useful in chronic cases, as the drug is kept in contact with the mucous membrane, and stronger applications can be used without danger, than can be employed when the lotion has to flow into the mouth and nose. In cases where the mucous membrane of the antrum is found to be much thickened, or even polypoid, great benefit is derived from freely scraping it. In order to carry this out properly a large opening must be made from the canine fossa. A very convenient form of sharp spoon is shown in the illustration. To prevent the risk of food being forced into the antrum during mastication, some kind of plate must be employed. The simplest, and most satisfactory, is a vulcanite plate fitted to the mouth, attached by clasps to the teeth, and having on its upper surface a spur or process of vulcanite, which fills the opening and projects very slightly into the antrum. The plate is removed in order to use the syringe. Plates are very commonly used provided with a tube (closed by a plug), through which syringing is carried out. This is much more difficult to keep clean, and offers no advantage over the simpler plate. When it is desired that the opening should close the spur is cut away, the plate being worn for a few days longer until healing is complete.

Plate provided with Spur.

Two possible sources of failure in treatment must be borne in mind (1) the cavity of the antrum may be partially divided by septa, rendering a thorough cleansing extremely difficult; (2) the root of a tooth may at some time have slipped into the cavity and becoming fixed to the wall, may by its presence prevent healing.

It is extremely difficult to say when the opening may be allowed to close, certainly not as long as there is any amount of discharge.

In conclusion, remembering how tedious these cases often prove, it is well to warn patients of this at the first, that they may not be disappointed if they do not immediately get relief.

## Figures and Tables

**Figure f1:**
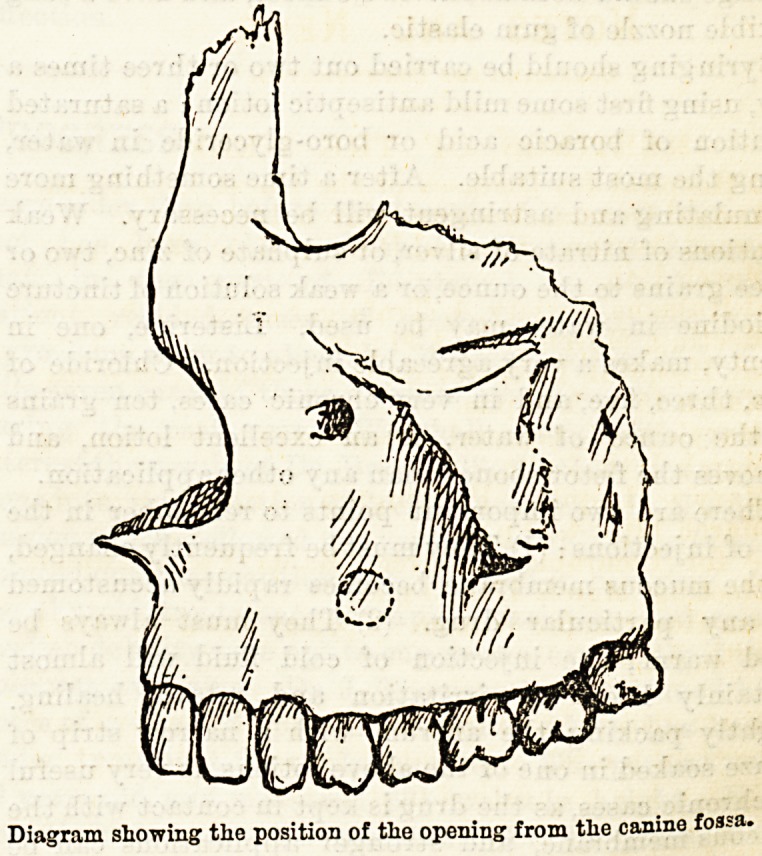


**Figure f2:**
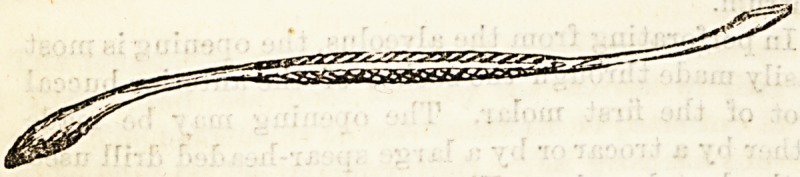


**Figure f3:**